# Nox1/4 inhibition exacerbates age dependent perivascular inflammation and fibrosis in a model of spontaneous hypertension

**DOI:** 10.1016/j.phrs.2020.105235

**Published:** 2020-11

**Authors:** R. Nosalski, T. Mikolajczyk, M. Siedlinski, B. Saju, J. Koziol, P. Maffia, T.J. Guzik

**Affiliations:** a Institute of Cardiovascular and Medical Sciences, University of Glasgow, Glasgow, UK; b Department of Internal and Agricultural Medicine, Faculty of Medicine, Jagiellonian University Medical College, Krakow, Poland; c Institute of Infection, Immunity and Inflammation, University of Glasgow, Glasgow, UK; d Department of Pharmacy, University of Naples Federico II, Naples, Italy

**Keywords:** SHR, GKT137831, NOX4, Perivascular inflammation, Ageing, Hypertension

## Abstract

Hypertension is associated with oxidative stress and perivascular inflammation, critical contributors to perivascular fibrosis and accelerated vascular ageing. Oxidative stress can promote vascular inflammation, creating options for potential use of NADPH oxidase inhibitors in pharmacological targeting of perivascular inflammation and its consequences.

Accordingly, we characterized age-related changes in oxidative stress and immune cell infiltration in normotensive (WKY) and spontaneously hypertensive rats (SHRs). Subsequently, we used pharmacological inhibitors of Nox1 (ML171) and Nox1/Nox4 (GKT137831; 60 mg/kg), to modulate NADPH oxidase activity at the early stage of spontaneous hypertension and investigated their effects on perivascular inflammation and fibrosis.

**Results:**

Ageing was associated with a progressive increase of blood pressure as well as an elevation of the total number of leukocytes, macrophages and NK cells infiltrating perivascular adipose tissue (PVAT) in SHRs but not in WKY. At 1 month of age, when blood pressure was not yet different, only perivascular NK cells were significantly higher in SHR. Spontaneous hypertension was also accompanied by the higher perivascular T cell accumulation, although this increase was age independent. Aortic Nox1 and Nox2 mRNA expression increased with age only in SHR but not in WKY, while age-related increase of Nox4 mRNA in the vessels has been observed in both groups, it was more pronounced in SHRs. At early stage of hypertension (3-months) the most pronounced differences were observed in Nox1 and Nox4. Surprisingly, GKT137831, dual inhibitor of Nox1/4, therapy increased both blood pressure and perivascular macrophage infiltration. Mechanistically, this was linked to increased expression of proinflammatory chemokines expression (CCL2 and CCL5) in PVAT. This inflammatory response translated to increased perivascular fibrosis. This effect was likely Nox4 dependent as the Nox1 inhibitor ML171 did not affect the development of spontaneous hypertension, perivascular macrophage accumulation, chemokine expression nor adventitial collagen deposition.

In summary, spontaneous hypertension promotes ageing-associated perivascular inflammation which is exacerbated by Nox4 but not Nox1 pharmacological inhibition.

## Graphical abstract

**Figure:**
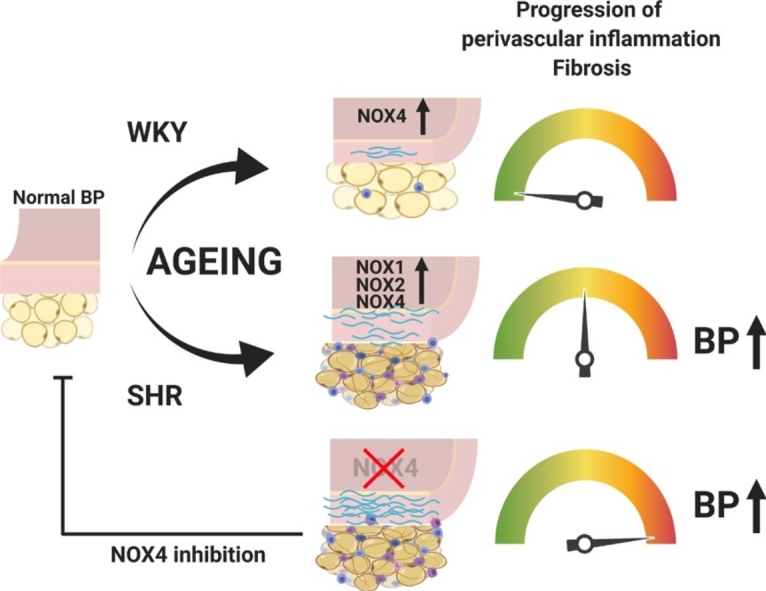

## 1 Introduction

Hypertension (HTN) is the leading cause of cardiovascular disease and premature death worldwide. One third of the population is hypertensive, while another third suffers from high normal blood pressure and commonly develops overt hypertension in subsequent years. Moreover, prevalence of HTN increases with age and is more than doubled in elderly than in young people [1,2]. Both clinical and mechanistic observations point to numerous similarities between mechanisms of ageing and hypertension, focused on early vascular ageing in hypertension [3]. Vascular senescence is related to increased vascular stiffening, fibrosis, and calcification and may be accelerated by HTN [3].

Perivascular adipose tissue (PVAT), which surrounds almost all blood vessels, plays a crucial role as a regulator of vascular homeostasis [4]. Aging is associated with low-grade perivascular inflammation and manifested by increased oxidative stress, augmented secretion of pro-inflammatory cytokines and chemokines, and intensified immune cell infiltration [5]. In the past several years it has become evident that the immune system plays an essential role in the development or sustaining of HTN. The role of T cells, B cells, macrophages and NK cells have been implicated in various models of experimental HTN [[6], [7], [8], [9], [10], [11], [12]] and in humans [13]. During the progression of HTN immune cells accumulate in target organs. These cells by releasing various pro-inflammatory mediators modify renal and vascular function, activating prohypertensive mechanisms and mediating end-organ damage. In the vasculature, key changes associated with perivascular inflammation include an increase in vascular resistance, fibrosis and endothelial dysfunction [4,14].

While inflammation has been recently implicated in the pathogenesis of hypertension, oxidative stress is recognised as a key mechanism of HTN and end-organ damage [15,16]. The predominant source of reactive oxygen species (ROS) are nicotinamide adenine dinucleotide phosphate (NADPH) oxidases (Nox). Nox enzymes are expressed throughout the vessel wall including in endothelial cells, vascular smooth muscle cells, fibroblasts, perivascular adipocytes as well as in infiltrating leukocytes. While the contribution of Nox1 and Nox2 homologues to experimental hypertension is well established, the role of Nox4 [17] remains disputed. Given the crucial role of Noxs in the pathogenesis of HTN, and their potential use as a therapeutic target, to date several Noxs inhibitors have been used in various *in vitro* and *in vivo* studies. Given the role of Nox1 and Nox4 in the process, the use of ML171 (2-acetylphenothiazine), the Nox1 inhibitor as well as GKT137831 (2-(2-chlorophenyl)-4-[3-(dimethylamino) phenyl]-5-methyl-1H-pyrazolo[4,3-c] pyridine-3,6(2H,5H)-dione), a dual inhibitor of Nox1 and Nox4 [18] are of particular interest. ML171 reduces vascular contractility in hypertensive rats or mice *ex vivo* [[19], [20], [21]]. GKT137831 attenuates cardiac remodelling and fibrosis in angiotensin II-induced HTN [22] and also reduces atherosclerosis, fibrosis and aortic inflammation in diabetic hyperlipidaemic apolipoprotein-E deficient mice [18].

Taking into account the well documented interactions between oxidative stress and inflammation [3,23], it creates a possibility that these inhibitors could be useful in reducing vascular inflammation and its consequences. However, their effects on blood pressure and/or vascular fibrosis in the context of perivascular inflammation has not yet been investigated to date.

Accordingly, we used a model of spontaneous hypertension and target organ damage to investigate the temporal changes of perivascular inflammation and fibrosis in the context of Nox enzyme expression [24,25] at different stages of the progression of hypertension. Finally, we investigated the effects of pharmacological inhibition of Nox4 and/or Nox1, on perivascular inflammation and perivascular fibrosis in spontaneous hypertension. Surprisingly, our data suggest that dual inhibition of Nox1 and Nox4 can accelerate early vascular ageing manifested by increased vascular fibrosis and perivascular inflammation in both normotensive and hypertensive rats.

## 2 Methods

### 2.1 Animals

Male spontaneously hypertensive rats (SHR/NCrl) and normotensive Wistar-Kyoto rats (WKYs) in four age groups were obtain from Charles River Laboratories and housed in controlled 12 h light/dark conditions, in a constant temperature of 21 °C, with ad libitum access to water and food. Part of the animals were randomly assigned to groups treated for 30 days with GKT137831 (CAS 1218942-37-0, Adooq Bioscience LLC, USA), ML171 (CAS 6631-94-3, Merck, Germany) or placebo mixed in food (both at 60 mg/kg/day [18]). Blood pressure was measured using non-invasive tail cuff plethysmography using BP2000 Blood Pressure Analysis System (Visitech Systems Inc.), following 7 days of training period as described before [26]. At the end of experiment, rats were heparinised and euthanized using CO_2_ inhalation and perfused via the left ventricle of the heart using cold PBS to ensure blood cells are not contributing to organ flow cytometric analysis. All *in vivo* work was performed in accordance with the Animals Scientific Procedures Act 1986 and ARRIVE (Animal Research: Reporting of In Vivo Experiments) Guidelines and approved by Jagiellonian University Ethics Committee (107/2014). All experiments conform the guidelines from Directive 2010/63/EU of the European Parliament on the protection of animals used for scientific purposes.

### 2.2 Isolation and characterisation of immune cells from adipose tissue

**Fig. 1:**
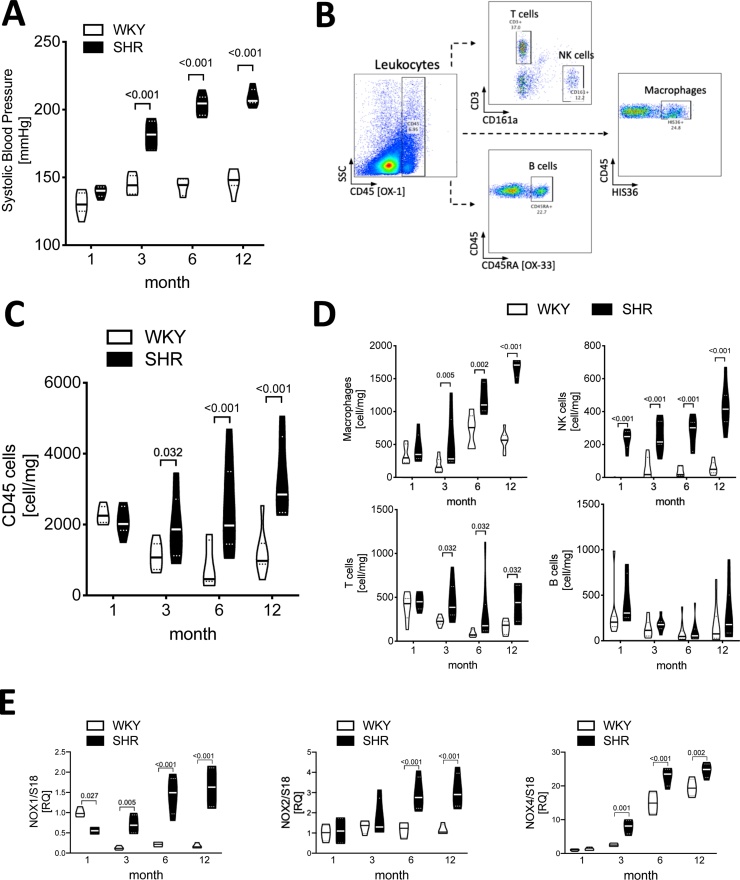
Temporal trends in perivascular inflammation and aortic NADPH oxidase expression level during ageing in spontaneously hypertensive and in normotensive rats. **A**. Blood pressure changes in 1-, 3-. 6- and 12-month old WKY and SHRs were measured by tail-cuff (n = 7/group). **B**. Representative density plots presenting the gating strategy of immune cells infiltrating perivascular adipose tissue studied by flow cytometry. **C**. Number of leukocytes (CD45+) infiltrating perivascular adipose tissue (cells/mg tissue; n = 7–9/group). **D**. Number of perivascular macrophages (HIS36+), NK cells (CD161+), T cells (CD3+) and B cells (OX-33+) (cells/mg tissue; n = 6–11/group). **E.** Aortic mRNA expression of NADPH oxidase subunits (Nox1, Nox2 and Nox4) (n = 8/group; 4 pooled samples of 2 animals/pool). Data presented on violin plots depicting median (**^__^**) and quartiles (^….^). Data analyzed using two-way ANOVA, p-values as indicated. Overall p values for two-way ANOVA, Panel **A** (p^BPxAgeing^ < 0.001, p^Ageing^ < 0.001, p^BP^ < 0.001), Panel **C** (p^BP x Time^ = 0.002, p^Time^ = 0.028, p^BP^ < 0.001), Panel **D**; macrophages (p^BP x Time^ < 0.001, p^Time^ < 0.001, p^BP^ < 0.001), NK cells (p^BP x Time^ = 0.008, p^Time^ < 0.001, p^BP^ < 0.001), T cells (p^BP x Time^ =< 0.49, p^Time^ =< 0.021, p^BP^ < 0.001), B cells (p^BP x Time^ =< 0.768, p^Time^ = 0.001, p^BP^ < 0.111), Panel **E**; Nox1 (p^Rat x Time^ < 0.001, p^Time^ = 0.007, p^Rat^ < 0.001), Nox2 (p^Rat x Tim^ = 0.018, p^Time^ = 0.01, p^Rat^ < 0.001), Nox4 (p^Rat x Time^ = 0.009, p^Time^ < 0.001, p^Rat^ < 0.001).

Perivascular adipose tissue was collected from aorta using dissection microscope. All lymph nodes were carefully removed. Subsequently, fat tissue was digested using collagenase type XI, collagenase IS and hyaluronidase (Sigma-Aldrich) dissolved in PBS containing calcium, magnesium and 20 μM HEPES for 30 min at 37 °C with regular agitation. The digested tissue was passed through a 70 μm sterile cell strainer (Falcon, BD Biosciences), to yield a single-cell suspension. Cells were then washed and resuspended in FACS buffer, counted, and stained with monoclonal antibodies for 30 min at 4 °C to distinguish the main immune subpopulations; leukocytes (CD45, clone OX-1, BioLegend), macrophages (clone His36, BD), NK cells (CD161a, clone 10/78, BD), T cells (CD3, clone 1F4, BD), B cells (CD45RA, clone OX-33, BD). Thereafter, cells were washed twice with FACS buffer and analyzed using flow cytometry (BD, FACSVerse). Data presented as an absolute number of cells isolated from perivascular adipose tissue normalized to the weight of the wet tissue (cell/mg) and percentage of the total CD45+ cells according to the gating strategy (Fig. 1B).

### 2.3 Gene expression analysis

Tissue was protected in RNALater stabilisation solution (Ambion) immediately after the harvest and next, RNA isolation was performed using RNeasy Mini Kit (Qiagen). Reverse transcription was performed using High Capacity cDNA Reverse Transcription Kit (Applied Biosystems, USA). Measurement of gene expression mRNA level was performed by real-time PCR system (7900 H T Applied Biosystem) using TaqMan probes; Rn00586652_m1 for *Nox1*, Rn00585380_m1 for *Nox4*, Rn00576710_m1 for *Nox2/cybb,* Rn00579590_m1 for *ccl5* and Rn00580555_m1 for *ccl2.* Eukaryotic 18S rRNA gene (Hs99999901_s1) was used as an internal control.

### 2.4 Histological staining

Formalin fixed and paraffin embedded 7 μm section of aorta with intact periaortic fat were deparaffinized and rehydrated. Aortic sections were stained with Weigert’s iron hematoxylin solution (Sigma-Aldrich) for 7 min, washed and incubated with 0.1 % Sirius red F3B (Sigma-Aldrich) for one hour in the dark. Slides were then washed in acidified water, dehydrated and mounted in DPX Mountant (Sigma-Aldrich). Quantification of adventitial collagen staining was performed using ImageJ software by blinded observers.

### 2.5 Statistical analysis

Statistical analysis was performed using GraphPad Prism 8.4.2. Data were analyzed using *t*-test, two-way ANOVA or repeated measures two-way ANOVA as indicated in figure legends. Shapiro–Wilk test was assessed to check normality of the data. Values of p < 0.05 were considered significant.

## 3 Results

### 3.1 Temporal trends in perivascular inflammation during ageing in spontaneous hypertension

One-month old rats did not show significant differences in the level of BP (Fig. 1A). Accumulation of leukocytes in PVAT was not overtly changed at 1 month of age (Fig. 1C–D), however, higher levels of NK cells were observed in SHRs (Fig. 1D). We then compared 1-month-old rats, in which the signs of pathology were not seen, with the 3-, 6- and 12-month-old animals. As expected, in 3-month-old SHRs a significant increase in BP was observed, reaching 180 ± 3 mmHg. Further increase was observed at 6-months and was maintained at 12-months of age in SHRs (203 ± 1 mmHg and 209 ± 1 mmHg, respectively) (Fig. 1A). No significant changes in BP levels were seen in control WKY rats at any age studied (Fig. 1A). Perivascular infiltration of leukocytes (CD45+) progressively increased starting from the 3rd month of age only in SHR, but not in control WKY rats (Fig. 1C). The most prominent accumulation in SHR PVAT was observed in relation to macrophages. Their number was significantly higher in SHR than WKY at 3rd month and continued to rise during ageing (Fig. 1D). Similar temporal trend was observed for NK cells in SHRs, but not in WKYs, (Fig. 1D). While the total number of T cells infiltrating PVAT was significantly higher in hypertensive, compared to normotensive rats at the 3rd, 6th and 12th month of age, no temporal change with age was observed (Fig. 1D). Finally, neither spontaneous hypertension nor ageing appeared to affect the presence of B cells in the PVAT (Fig. 1D).

### 3.2 The development of hypertension and ageing increases aortic NADPH oxidase expression

To further understand the role of oxidative stress in the regulation of perivascular inflammation, we studied the mRNA expression of vascular NADPH oxidase subunits. The progression of hypertension observed in 3-month-old SHRs, was associated with significant elevation of both Nox1 and Nox4 mRNA level in the aortas. Surprisingly, a significantly higher level of Nox1 mRNA was found in 1-month-old WKYs in comparison to SHRs (Fig. 1E) and this relationship was reversed at 3rd month of life. This could reflect a physiological role of oxidative stress at the earliest stages of life in the offspring animals. A 5-, 6- and 9-fold induction of Nox1 mRNA was observed in the vessels of 3-, 6- and 12-month-old SHRs, respectively. Interestingly Nox2 mRNA levels were 3-fold higher in the aortas from SHR than WKY only at the 6th and 12th month of life, after severe hypertension had already established (Fig. 1E middle panel). Vascular Nox4 mRNA increased with age in both SHR and WKY, this increase was more pronounced in SHRs compared to normotensive controls (p^int^<0.05).

### 3.3 Pharmacological inhibition of Nox1/Nox4 hastens the rise of BP and exacerbates perivascular inflammation

**Fig. 2:**
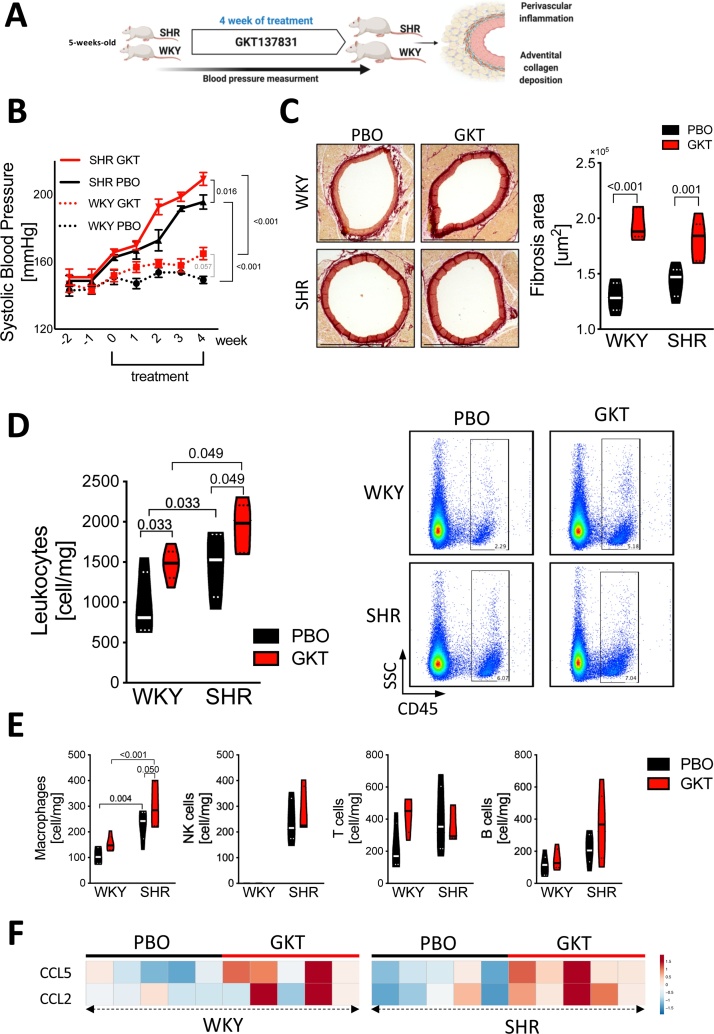
Pharmacological inhibition of Nox1/Nox4 hastens the rise of BP and exacerbates perivascular inflammation. **A**. Schematic depicting experimental design and workflow of Nox1/4 pharmacological inhibition. **B**. Blood pressure level in WKY and SHR treated with GKT137831 (GKT) or placebo (PBO) measured by tail-cuff. Data analyzed using repeated measures two-way ANOVA (n = 5/group) (p^Time x Treatment^ < 0.001, p^treatment^ < 0.001, p^time^ < 0.001). **C**. Perivascular collagen accumulation assessed by picosirius red staining (left panel, representative of n = 5/group, scale bar 1000 μm) and quantitative analysis of perivascular collagen deposition (right panel). **D**. Number of tissue infiltrating leukocytes (CD45+) in perivascular adipose tissue (panel left) with representative density plots studied by flow cytometry (right panel) (cells/mg tissue; n = 5/group). **E**. Numbers of macrophages (HIS36+), NK cells (CD161+), T cells (CD3+) and B cells (OX-33+) per mg of PVAT studied by flow cytometry (n = 5/group). Data presented on violin plots; (**^__^**) depicting median and (^….^) depicting quartiles. Data analyzed using two-way ANOVA, p-values as indicated. **F**. Heatmaps presenting mRNA expression level of chemokine RANTES (*CCL5)* and MCP-1 (*CCL2)* in perivascular adipose tissue collected from PBO or GKT137831 treated rats. Data analyzed by *t*-test (WKY PBO vs WKY GKT137831; *CCL5* p = 0.01, *CCL2* p = 0.26 and SHR PBO vs. SHR GKT137831; *CCL5* p = 0.007, *CCL2* p = 0.02). Overall p values for two-way ANOVA; for Panel B (p^Rat x Treatment^ = 0.88, p^Rat^ = 0.006, p^Treatment^ = 0.006), Panel C; macrophages (p^Rat x Treatment^ = 0.57, p^Rat^ < 0.001, p^Treatment^ = 0.028), DC (p^Rat x Treatment^ = 0.34, p^Rat^ = 0.82, p^Treatment^ = 0.001), NK cells (p^Rat x Treatment^ = 0.47, p^Rat^<0.001, p^Treatment^ = 0.47), T cells (p^Rat x Treatment^ = 0.08, p^Rat^ = 0.33, p^Treatment^ = 0.22), B cells (p^Rat x Treatment^ = 0.39, p^Rat^ = 0.013, p^Treatment^ = 0.127).

As both Nox1 and Nox4 mRNA was increased in SHRs in a particular age dependent fashion, we next used GKT137831, a dual Nox1/4 inhibitor, in both normotensive and hypertensive animals. We started therapy at early stage of pathology (5 weeks of age; Fig. 2A) development and investigated its effects on perivascular immune cell infiltration and fibrosis as well as on hypertension development at this early progressive phase. GKT137831 showed a significant (p^treatment^ < 0.001) global effect on the rise of BP in treated rats. While the animals showed similar BP levels at the beginning of the experiment, Nox1/4 inhibition significantly accelerated the development of HTN in SHRs, which after 4 weeks of treatment was significantly higher (Fig. 2B). Interestingly, the spontaneous increase in BP was observed one week earlier in GKT137831 treated rats than in placebo treated SHRs, and later BP level remained higher throughout the whole experiment. Nox1/4 inhibition showed a similar trend in normotensive rats, where BP modestly increased after the GKT137831 treatment (p = 0.057).

Next we used picrosirius red staining to evaluate adventitial collagen deposition. GKT137831 treatment dramatically escalated the fibrosis area around the aortas in both normotensive and hypertensive rats (p^treatment^ < 0.001) (Fig. 2C).

As we and others have recently demonstrated the key role of perivascular inflammation in mediating perivascular collagen deposition, we next investigated leukocyte infiltration in perivascular fat in studied groups. GKT137831 treatment significantly (p^treatment^ = 0.006) exacerbated perivascular immune cell infiltration in SHRs and surprisingly also in WKYs rats when compared with placebo-treated groups (Fig. 2D). Further studies showed that this increase was particularly linked to increased number of macrophages (p^treatment^ < 0.028) infiltrating the perivascular adipose tissue (Fig. 2E). No significant changes were observed with respect to NK cell, T cells and B cells in the context of Nox1/Nox4 inhibition (Fig. 2E).

Perivascular recruitment of immune cells are controlled primarily by chemokines [27], therefore in order to investigate possible mechanism of leukocyte expansion upon Nox1/4 inhibition, we evaluated the mRNA expression of C-C Motif Chemokine Ligand 2 (CCL2) and CCL5. While, GKT137831 significantly increased CCL5 mRNA expression in both WKYs (p = 0.013) and SHRs, (p = 0.021), CCL2 mRNA levels were significantly elevated only in hypertensive animals (p = 0.039) when compared with placebo-treated rats (Fig. 2F).

### 3.4 Pharmacological inhibition of Nox1 has modest effect on blood pressure and perivascular inflammation

**Fig. 3:**
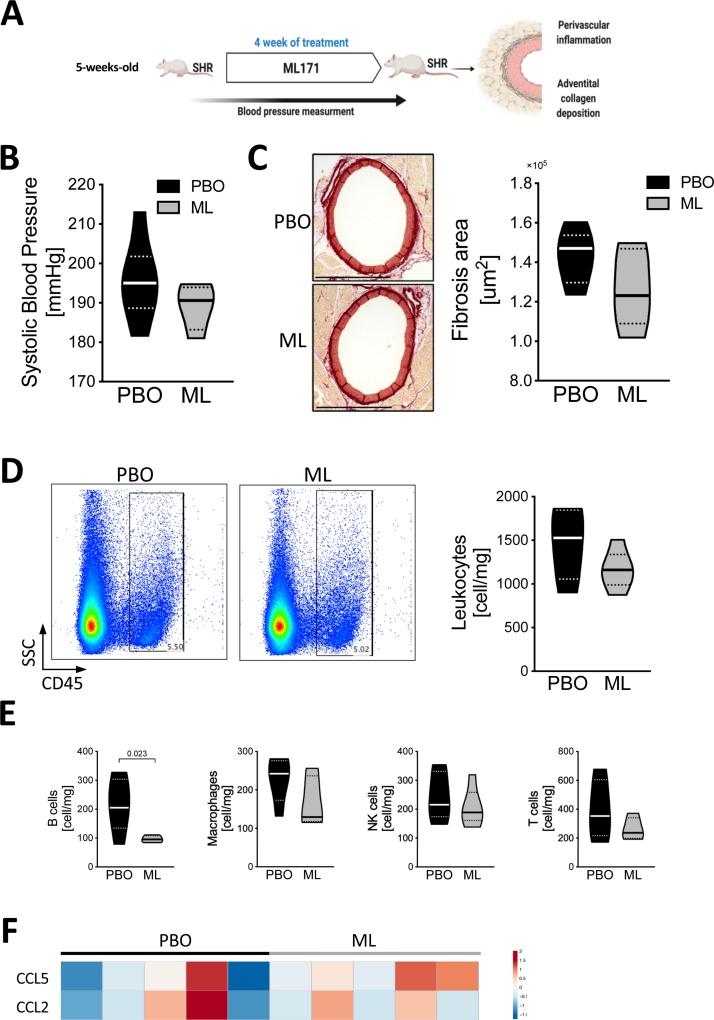
Pharmacological inhibition of Nox1 has modest effect on blood pressure and perivascular inflammation. **A**. Schematic of the experimental design and workflow of pharmacological inhibition of Nox1. **B**. Blood pressure level in SHR treated with PBO or ML171 (ML) measured by tail-cuff. **C**. Perivascular collagen accumulation assessed by picrosirius red staining (left panel, representative of n = 5/group, scale bar 1000 μm) and quantitative analysis of perivascular collagen deposition (right panel). **D**. Total number of leukocytes (CD45+) infiltrating perivascular adipose tissue (right panel) with representative density plots studied by flow cytometry (left panel) (n = 5/group). **E**. Number of B cells (OX-33+), macrophages (HIS36+), NK cells (CD161+) and T cells (CD3+) per mg of PVAT studied by flow cytometry (n = 5/group). Data presented on violin plots; (**^__^**) depicting median and (^….^) depicting quartiles. Data analyzed using *t*-test, p-values as indicated. **F**. Heatmap presenting mRNA expression level of chemokine RANTES (*CCL5*) and *MCP-1* (*CCL2*) in perivascular adipose tissue collected from PBO and ML171 treated rats. Data analyzed by *t*-test (CCL5 p = 0.2; CCL2 p = 0.9; PBO vs ML171, n = 5/group).

As GKT137831 has been shown to inhibit both Nox4 and Nox1, we next investigated effects of selective Nox1 inhibition using ML171 for 4 weeks in 5-week old SHRs compared to placebo (Fig. 3A). The experiment was not performed in WKY as mRNA levels of Nox1 remained borderline undetectable in WKY throughout all studied age range. In contrast to GKT137831, ML171 treatment resulted in modest reduction of blood pressure (p = 0.2) (Fig. 3B). Similarly, there was no significant reduction in adventitial collagen deposition (p = 0.18), observed in ML171 treated rats (Fig. 3C). Moreover, pharmacological inhibition of Nox1 did not affect the total number of leukocytes (Fig. 3D), macrophages, T cells, or NK cells (Fig. 3E) infiltrating the perivascular adipose tissue. In contrast, ML171-treated rats showed lower accumulation of B cells around the vasculature in comparison to placebo-treated SHRs. Finally, in contrast to double Nox4/Nox1 inhibition, Nox1 inhibitor did not affect the CCL2 and CCL5 mRNA expression (Fig. 3F).

## 4 Discussion

Inflammation and oxidative stress have been implicated in the pathogenesis of hypertension. However, the interrelationships between these two phenomena are not well defined. Moreover, ageing as the main risk factor for cardiovascular disease can potentiate not only BP increase but also inflammation and reactive oxygen species generation leading to end-organ damage such as vascular stiffness [1,3,27]. In the current study, we have studied effects of ageing on perivascular inflammation in a model of spontaneous hypertension. Moreover, using a pharmacological manipulation, we have highlighted the importance of Nox1 and Nox4 in the modulation of perivascular inflammation and vascular fibrosis as well as BP increase regulation.

We used spontaneously hypertensive rats (SHR), which are the most comprehensive model of essential hypertension. Similarly to humans, the etiology of spontaneous hypertension follows the same progression of HTN with pre-hypertensive phase, developing phase, and sustained hypertension phase [24]. According to our knowledge, the systematic comparison of perivascular inflammation and vascular NADPH oxidases expression in vasculature of these rats has not been performed, to date. Accordingly, we compared 1-, 3-, 6- and 12-month old male hypertensive rats indicating pre-hypertensive phase, development stage, and two last time-point phases when the hypertension was well established. As expected, the young SHRs showed similar systolic BP compared with WKY rats. This is consistent with many reports where hypertension development is observed after 6–8 weeks of age [24]. While in humans ageing is a risk factor for the development of hypertension, our current observations and other [28,29] studies did not show changes in BP level in WKY rats, suggesting that longer follow up may be required.

In the past several years it has become evident that the immune system plays an essential role in the development of hypertension. Studies using numerous models of genetically modified immune responses as well as using pharmacological blockade of immune cell interactions, show a causal role of immune system in blood pressure increases caused by various pro-hypertensive stimuli [4,11]. These studies have been recently supported by Mendelian randomization analysis in UK biobank population confirming causal link between T cells and blood pressure and hypertension [13]. During hypertension progression, immune cells accumulate in target organs, particularly around the vasculature in PVAT or adventitia [14] leading to perivascular inflammation.

Consistent with previous studies coming from angiotensin II (Ang II)-induced or DOCA-salt models of hypertension [4,26], we showed that in accordance with BP elevation, spontaneous hypertension was associated with the presence of perivascular inflammation. Interestingly, we observed that at 1-month of age, while animals are still normotensive only NK cells (CD161+) are significantly increased in PVAT, while the total number of leukocytes, macrophages, T cell and B cells infiltrating the perivascular fat were, in fact, comparable between very young SHRs and WKY rats. The observation of a potential role of NK cells is consistent with previous reports of increased proportion of CD161+ cells in the spleen of WKY and SHR strains which we have now extended to local tissue directly relevant for hypertension pathology [30]. Interestingly, this can indicate that higher number of these cells in PVAT, known to release cytokines such as IFN-γ, TNF-α or cytotoxic mediators like granzyme, could trigger the chronic low-grade inflammation and be responsible for the initiation of a pro-hypertensive phenotype. During ageing, in parallel with the BP elevation, a higher number of leukocytes infiltrating PVAT was observed only in SHRs, but not in WKY rats. While spontaneous hypertension was linked with higher number of almost all immune cell types, excluding B cells, age-related increases were observed only among macrophages and NK cells, suggesting their role in the progression of spontaneous hypertension in rats. However, T cell derived pro-inflammatory cytokines or B cells derived autoantibodies have been shown to play a crucial role in other models of hypertension [31].

While the role of immunity and inflammation has been confirmed by hundreds of studies now, optimal methods to inhibit inflammation are difficult to develop, as we need to avoid overt immune suppression.

As oxidative stress is a key feature of hypertension and is known to regulate redox dependent inflammatory molecules [32], better understanding of the interplay between inflammation and oxidation in hypertension may provide an opportunity for safe and efficient way to target immune and inflammatory responses in hypertension. This concept is supported by observations that mice lacking NADPH oxidase subunits are protected against hypertension and perivascular inflammation [4]. However, the role of Nox1 and Nox4 in the context of ageing, development of spontaneous hypertension and in modulation of the perivascular inflammation has not been investigated to date.

In the present study, we confirmed the increases in the expression of Nox1, Nox2 and Nox4 during the development of spontaneous hypertension. The profile of temporal changes of these molecules with age in SHR showed that Nox1 and Nox4 are increased already in the developing stages of hypertension (3rd month), while Nox2 upregulation was observed only in sustained hypertension (beyond 6th month of age), that might suggest a less pronounced role in the initiation of the pathology. Such increase of Nox2, could also be related to pronounced infiltration of Nox2 expressing immune cells infiltrating adventitia and PVAT, rather than increased expression by vascular cells. The perivascular nature of immune cell infiltration in hypertension is important as well as the fact that the overall age-related increases of Nox enzymes in the vasculature occurred in parallel to increased PVAT inflammation.

Given that Nox1 was the most upregulated NADPH oxidase homologue in the aortas of hypertensive rats and also age-related increase was observed only in SHRs, we decided to assess whether Nox1 inhibition affects BP elevation and perivascular inflammation. However, treatment of pre-hypertensive rats with the selective Nox1 inhibitor ML171 did not affect the development of spontaneous hypertension and had only a minor effect against perivascular leukocytes accumulation and chemokines level changes or adventitial fibrosis. These results are not consistent with the data coming from Nox1^−/−^ mice subjected to Ang II-induced hypertension [33]; however, this finding could also suggest that Nox1 is more important in the Ang II model rather than in SHR rats.

The rise in BP of hypertensive animals was also associated with significant induction of both Nox1 and Nox4 mRNA expression in the aortas. However, despite a significant increase was observed in hypertensive rats, an age-related escalation of Nox4 mRNA expression was also demonstrated in the vessels of normotensive animals, but this increase was more dynamic in SHRs. This could suggest that the increment of Nox4 mRNA level may constitute the presence of a compensatory mechanism occurring during the ageing. Inhibition of both Nox1/Nox4 resulted in an accelerated elevation of BP and higher collagen deposition in the aortas observed in both, SHRs and WKY rats. It is widely accepted that ageing and hypertension lead to an increase in arterial fibrosis. Interestingly the profibrotic effect of GKT137831 was observed in both WKY and SHR. This is important also in the context of the fact that 20-month-old WKY rats, despite the lack of BP elevation show increased aortic stiffness as well as SHRs with well-established HTN [28]. Thus, our observations are consistent with GKT137831 promoting accelerated vascular ageing in both, normotensive and hypertensive animals. Protective role of Nox4 has been demonstrated in various models in relation to vascular pathology [34]. Nox4 is the main source of hydrogen peroxide (H_2_O_2_) in the vasculature which plays an important role as a signalling molecule controlling gene expression, cells differentiation and by driving pro-survival signalling protective against ageing [35,36]. It can act in a dose-dependent manner mediating physiological and pathophysiological processes in cardiovascular diseases [37,38]. Hydrogen peroxide derived from Nox4 in the vasculature maintains the expression of eNOS [39] and directly promotes vascular relaxation [37] contributing to the regulation of vascular tone.

We have recently shown that vascular fibrosis is regulated by perivascular inflammation and increased immune cell recruitment into PVAT [26]. Indeed, GKT137831 treatment increased fibrosis and accumulation of leukocytes, particularly macrophages, in the PVAT of both normotensive and hypertensive rats. Furthermore, this process was related to a higher level of inflammatory chemokine expression (CCL2 and CCL5) in PVAT, whose crucial role in hypertension and other CVDs is well established [27,[40], [41], [42], [43], [44]]. It has been shown that combined Nox1/4 inhibition with GKT137831 reduces vascular leukocytes infiltration in atherosclerosis [45], or suppresses proinflammatory and profibrotic processes in a model of diabetic nephropathy [46], both results are contrary to our study. However, GKT137831 mimicked the effect of Nox1^−/−^, while Nox4 deficiency did not promote anti-inflammatory and anti-fibrotic effects in these studies. In our current study, the effects seem to be mediated more through Nox4 than Nox1. Furthermore, there are reports showing that GKT127831 increases E-selectin expression and adhesion of immune cells [47], promoting perivascular inflammation. Together, these results suggest that the role of Nox4 is complex and inhibition of Nox1/4 could drive different actions in various pathological diseases.

While our study showed that potential adverse effects of Nox4/Nox1 pharmacological inhibition are pronounced in the vasculature in hypertension, the current study did not establish a clear answer to the problem of the relationship between perivascular inflammation and BP elevation in SHR. NK cells emerge as a possible early mediator of this process in SHR rats. Furthermore, it remains unclear whether vascular oxidative stress or perivascular inflammation are primary in the development of spontaneous hypertension. While more mechanistic studies are needed, our results indicate that NK cells might be an early mediator of this process in SHR rats. They are capable of releasing pro-inflammatory cytokines which can modulate vascular NADPH activity or/and regulate chemokines expression in the vasculature further fuelling subsequent inflammation.

The study has several limitations that need to be acknowledged. We did not, for example, focus on measures of oxidative stress as these measurements have been widely reported before. Instead, we postulated that NADPH oxidase targeting could affect perivascular inflammation. Secondly, the use of pharmacological inhibitors has inherent limitation related to their specificity, limiting mechanistic insights that can be gained. Nevertheless, it does not diminish the value of defining vascular effects of these pharmacological substances, the use of which has been postulated for upcoming clinical trials.

**Fig. 4:**
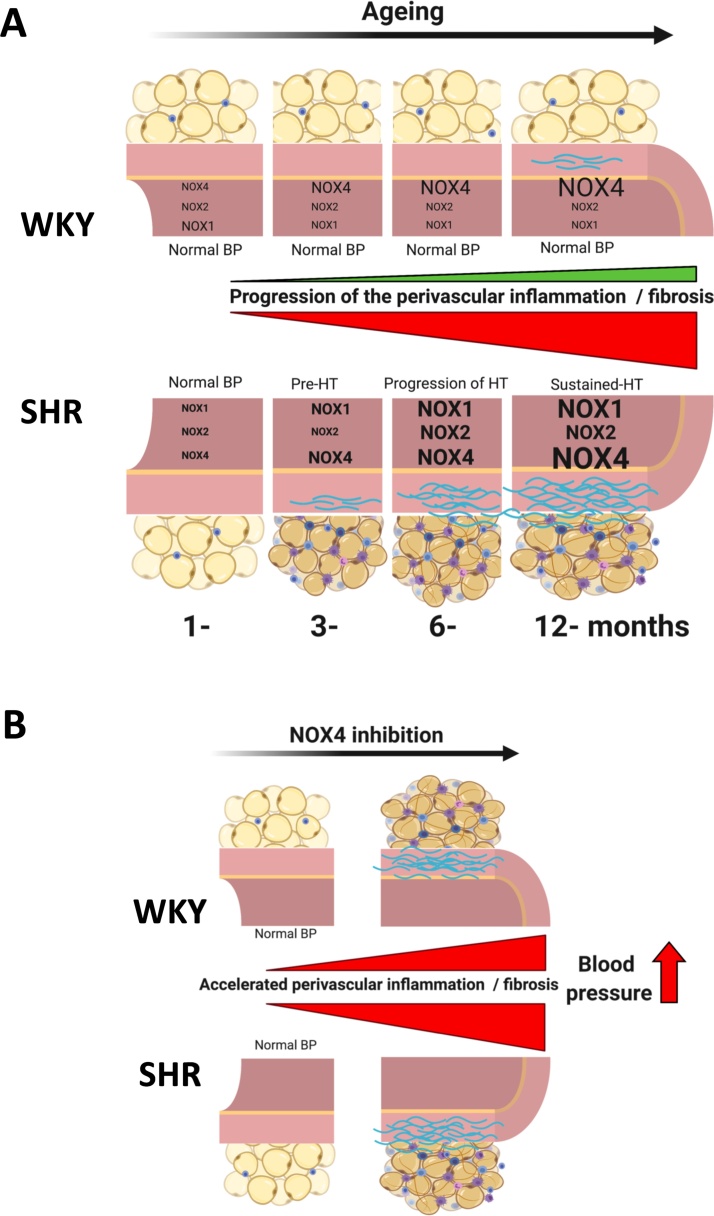
Summary of the effects of ageing (A) and pharmacological inhibition of Nox1/Nox4 on studied vascular phenotypes. **A**. Ageing in spontaneously hypertensive rats (SHR) is associated with a progressive increase in blood pressure, exacerbation of perivascular inflammation and elevation of mRNA expression of vascular NADPH oxidase subunits. While these effects are barely visible in normotensive rats (WKY). **B**. Pharmacological inhibition of Nox1/4, using GKT137831, increase blood pressure, hastens accumulation of immune cell accumulation in PVAT and escalate perivascular collagen deposition leading to accelerated vascular ageing in both normotensive (WKY) and hypertensive rats (SHR).

In summary, ageing and spontaneous hypertension are associated with a progressive increase in both blood pressure and perivascular inflammation (Fig. 4A). In contrast to our initial hypothesis, inhibition with pharmacological inhibitors of Nox1/4 hasten these features of accelerated vascular ageing, while inhibition of Nox1 exerts only minor effects on perivascular inflammation or development of spontaneous hypertension in rats (Fig. 4B). These effects need to be taken into account in designing future antioxidant strategies targeting NADPH oxidases in hypertension.

## Declaration of Competing Interest

None.
